# Squeezing water from a stone: high-throughput sequencing from a 145-year old holotype resolves (barely) a cryptic species problem in flying lizards

**DOI:** 10.7717/peerj.4470

**Published:** 2018-03-20

**Authors:** Jimmy A. McGuire, Darko D. Cotoras, Brendan O’Connell, Shobi Z.S. Lawalata, Cynthia Y. Wang-Claypool, Alexander Stubbs, Xiaoting Huang, Guinevere O.U. Wogan, Sarah M. Hykin, Sean B. Reilly, Ke Bi, Awal Riyanto, Evy Arida, Lydia L. Smith, Heather Milne, Jeffrey W. Streicher, Djoko T. Iskandar

**Affiliations:** 1 Museum of Vertebrate Zoology, University of California, Berkeley, CA, United States of America; 2 Department of Integrative Biology, University of California, Berkeley, CA, United States of America; 3 Department of Ecology and Evolutionary Biology, University of California, Santa Cruz, CA, United States of America; 4 Entomology Department, California Academy of Sciences, San Francisco, CA, United States of America; 5 Department of Biomolecular Engineering and Bioinformatics, Baskin School of Engineering, University of California, Santa Cruz, CA, United States of America; 6 Jalan Hayam Wuruk, United in Diversity Foundation, Jakarta, Indonesia; 7 Key Laboratory of Marine Genetics and Breeding, Ocean University of China, Qingdao, China; 8 Department of Environmental Science, Policy, and Management, University of California, Berkeley, CA, United States of America; 9 Computational Genomics Resource Laboratory, California Institute for Quantitative Biosciences, University of California, Berkeley, CA, United States of America; 10 Museum Zoologicum Bogoriense, Research Center for Biology-The Indonesian Institute of Sciences, Cibinong, Indonesia; 11 Department of Life Sciences, The Natural History Museum, London, United Kingdom; 12 School of Life Sciences and Technology, Institute of Technology Bandung, Bandung, West Java, Indonesia; 13 Current affiliation: Center for Comparative Genomics, California Academy of Sciences, San Francisco, CA, United States of America; 14 Current affiliation: Department of Medical & Molecular Genetics, School of Medicine, Oregon Health & Science University, Portland, OR, United States of America

**Keywords:** *Draco*, Formalin-fixation, Ancient DNA, Phylogeny, Taxonomy

## Abstract

We used Massively Parallel High-Throughput Sequencing to obtain genetic data from a 145-year old holotype specimen of the flying lizard, *Draco cristatellus*. Obtaining genetic data from this holotype was necessary to resolve an otherwise intractable taxonomic problem involving the status of this species relative to closely related sympatric *Draco* species that cannot otherwise be distinguished from one another on the basis of museum specimens. Initial analyses suggested that the DNA present in the holotype sample was so degraded as to be unusable for sequencing. However, we used a specialized extraction procedure developed for highly degraded ancient DNA samples and MiSeq shotgun sequencing to obtain just enough low-coverage mitochondrial DNA (721 base pairs) to conclusively resolve the species status of the holotype as well as a second known specimen of this species. The holotype was prepared before the advent of formalin-fixation and therefore was most likely originally fixed with ethanol and never exposed to formalin. Whereas conventional wisdom suggests that formalin-fixed samples should be the most challenging for DNA sequencing, we propose that evaporation during long-term alcohol storage and consequent water-exposure may subject older ethanol-fixed museum specimens to hydrolytic damage. If so, this may pose an even greater challenge for sequencing efforts involving historical samples.

## Introduction

The advent of Massively Parallel High-Throughput Sequencing (HTS) has dramatically altered the manner in which geneticists conduct their research. This is certainly true for molecular phylogeneticists and population geneticists, who now routinely have access to large multilocus genetic datasets for non-model organisms. Because HTS using the Illumina platform involves sequencing of small fragments of DNA, this approach offers the potential to access previously unattainable genome-scale sequence data even for degraded historical samples (e.g., Prüfer et al., 2014; Palkopoulou et al., 2015). Millions of fluid-preserved specimens in museum collections predate the development of allozyme and DNA sequencing technologies, and thus lack specially preserved tissue samples for genetic analysis. Formalin-fixed fluid specimens, usually having highly fragmented and cross-linked DNA, are often refractory to sequencing efforts using traditional Sanger sequencing. However, recent studies have shown that it is possible to obtain genomic DNA sequences from some of these fluid-preserved museum specimens. Hykin, Bi & McGuire (2015) demonstrated that low-coverage genomic sequences could be recovered from a 30-year old formalin-fixed museum specimen, though they were unsuccessful with a ∼100-year old specimen. Ruane & Austin (2016) sequenced Ultra-Conserved Elements (UCEs) from both formalin-fixed (*n* = 11) and ethanol-fixed (*n* = 10) museum specimens, including one sample that was collected between 1878 and 1911. Both had mixed success, with the quantity of DNA recovered in the extraction stage likely playing the largest role in the performance of their sequencing efforts. Notably, the samples that failed in the Ruane & Austin (2016) experiment included subsets of both their formalin- (seven of 16) and alcohol-fixed (four of five) samples, indicating that old alcohol-preserved museum specimens are not necessarily less problematic than those initially fixed with formalin. This is surprising given that contemporary tissue samples earmarked for genetic analysis are routinely stored in 95% ethanol.

Our objective in this study was to address an otherwise intractable problem in flying lizard taxonomy using Illumina HTS and ancient DNA methods for a 145-year old fluid-preserved holotype specimen. The nettlesome taxonomic issue involves a small clade of poorly known flying lizards (genus *Draco*, Agamidae) that, for reasons outlined below, was unlikely to be resolved without obtaining genetic data from the holotype specimen of one of the constituent species, *Draco cristatellus*. Determining species limits within this small clade (the *Draco fimbriatus* group) has proven challenging for taxonomists, and we first describe the convoluted taxonomic history of the clade as a justification for our ultimate solution to this question involving HTS. The *D. fimbriatus* group currently includes four recognized species: *D. abbreviatus*, *D. cristatellus, D. fimbriatus*, and *D. maculatus*. This taxonomic framework is based on Manthey (2008) and was followed by the widely utilized Reptile Database (Uetz, 2006). For reasons that we will describe in a subsequent paper, we instead utilize an alternative taxonomy that includes *D. cristatellus*, as well as *D. fimbriatus* (=*D. abbreviatus* above), *D. hennigi*, *D. punctatus* (=*D. fimbriatus* above), and *D. maculatus.* We note that our taxonomy differs from that of Manthey (2008) primarily as a consequence of having information that indicates that the type locality of *D. fimbriatus* is the Malay Peninsula rather than Java. We further note that our recognition of both *D. cristatellus* and *D. punctatus* is tentative, as a primary objective of this paper is to resolve whether these are in fact distinct species.

The *Draco fimbriatus* group is taxonomically challenging. Although *Draco maculatus* is abundant, easily sampled in the field, and easily distinguished from other members of the group based on external phenotype, the remaining members of this clade are only rarely encountered, with relatively few specimens represented in museum collections. These species are canopy specialists (McGuire, 2003), making them more difficult to detect and more challenging to collect than other *Draco* taxa. Furthermore, the species comprising the *D. fimbriatus* group, as well as several other *Draco* clades are primarily distinguished on the basis of differences in coloration of their display structures (dewlap and patagia for most *Draco* taxa, just the dewlap among the relevant members of the *D. fimbriatus* group). For example, two major clades—the ‘Philippines *volans* group’ (McGuire & Alcala, 2000) and the ‘*Draco lineatus* group’ (McGuire et al., 2007)—are each composed of multiple species that are primarily distinguished on the basis of coloration. Because coloration fades in preservative, recognizing species-specific coloration characteristics generally requires experience with the species in the field and/or access to color imagery of the specimen in life. Thus, as museum specimens, the members of these clades can become functionally cryptic sympatric species. In summary, for the *D. fimbriatus* group, the paucity of museum specimens, and the rarity with which specimens are observed in the field from throughout their collective ranges by single observers, has greatly impeded taxonomic progress.

Within the *Draco fimbriatus* group, a particularly challenging issue relates to the taxonomic standings of *D. cristatellus* Günther, 1872 and *D. punctatus* Boulenger, 1900. *Draco cristatellus* was described based on a single specimen collected by Mr. Alfred Hart Everett in Matang, Sarawak between 1869 and March of 1872 (when Günther’s manuscript describing the species was submitted for publication). Although Everett collected the type specimen, the Trustees of the British Museum purchased it from Mr. W. Cutter, thereby making it available to Günther for description (see Günther, 1872). Because Günther presumably did not see the living specimen, he did not evaluate the coloration of the dewlap in life, which is essential for species identification within this group. Günther described the dewlap as ‘golden-yellow, with a brown anterior edge’, presumably from its preserved state. Subsequently, Boulenger (1900) described *D. punctatus* from Bukit Larut on the Malay Peninsula, noting that the dewlap was lemon yellow in coloration. Although he did not attempt to diagnose *D. punctatus* from *D. cristatellus*, Boulenger (1900) was clearly aware of the latter species and explicitly considered his *D. punctatus* holotype to be taxonomically distinct. Indeed, Boulenger (1900) noted that he had examined a second specimen of *D. punctatus* from Sarawak that was also collected by Everett, remarking that he had previously referred that second specimen to *D. cristatellus*. Boulenger (1900) might be the last author to have had a clear idea about the taxonomic distinctiveness of *D. cristatellus* and *D. punctatus,* and it is a pity that he did not identify the character differences that he used to render his taxonomic decision. Although De Rooij (1915) recognized *D. fimbriatus*, *D. cristatellus* and *D. punctatus* as distinct species, subsequent authors synonymized one or more members of the group. Hennig (1936) synonymized *D. cristatellus* with *D. fimbriatus*, while continuing to recognize *D. punctatus.* In his monographic *Draco* taxonomic study, Musters (1983) opted to synonymize both *D. cristatellus* and *D. punctatus* with *D. fimbriatus*. In his competing taxonomic treatment, Inger (1983) recognized two species, *D. cristatellus* and *D. fimbriatus,* as valid species, but placed *D. punctatus* in the synonymy of *D. cristatellus*. Inger’s (1983) recognition of two species was based in part on Grandison’s (1972) report on two sympatric *D. fimbriatus* group species with distinct dewlap colorations on Gunung (Mt.) Benom on the Malay Peninsula. Whereas Grandison (1972) identified the two sympatric species as *D. fimbriatus* and *D. punctatus* (without commenting on the status of the Bornean *D. cristatellus*), Inger (1983) instead opted to treat *D. punctatus* as a synonym of *D. cristatellus*. This sensible decision was presumably made on the basis of the similar dewlap colorations of the *D. cristatellus* and *D. punctatus* holotypes (‘golden-yellow, with a brown anterior edge’ vs. ‘lemon yellow’). Inger (1983) furthermore attempted to differentiate his conceptions of *D. fimbriatus* and *D. cristatellus* using a statistical analysis of eight linear measurements and scale counts. Although he successfully sorted his sample into two groups on the basis of overlapping but significantly distinct character state differences, his *a priori* placement of *D. punctatus* in the synonymy of *D. cristatellus* effectively precluded the possibility that three species –*D. cristatellus, D. fimbriatus*, and *D. punctatus*—might all co-occur on the Greater Sunda Shelf (and particularly on Borneo). Here we address this open question taking advantage of two critical developments: (1) the acquisition and analysis of a key specimen (TNHC 56763) obtained by JAM from Santubong, Sarawak, Malaysian Borneo in 1996, and (2) an analysis of the 145-year old *D. cristatellus* holotype using ancient DNA extraction methods and HTS on the Illumina platform. We establish the following hypothesis-testing framework: In Hypothesis 1, *D. cristatellus* and *D. punctatus* are synonyms, together representing a single species distinct from *D. fimbriatus* and TNHC 56763. In Hypothesis 2, *D. cristatellus* and *D. fimbriatus* are synonyms. In Hypothesis 3, *D. cristatellus* is a species distinct from *D. punctatus* and *D. fimbriatus* but conspecific with TNHC 56763. Finally, in Hypothesis 4, *D. cristatellus*, *D. fimbriatus*, *D. punctatus*, and TNHC 56763 all represent distinct species, with TNHC 56763 representing a fourth sympatric species on Borneo. The only way to conclusively test these alternative hypotheses is to obtain informative genetic data from the holotype specimen of *D. cristatellus* for comparison with TNHC 56763, as well as representative specimens of *D. fimbriatus* and *D. punctatus*.

## Materials and Methods

### (a) DNA extraction and sequencing from the *Draco cristatellus* holotype

We obtained from the Natural History Museum in London liver tissue from the holotype of *Draco cristatellus* (specimen BMNH 1872.2.19.4). This specimen was originally collected and prepared prior to March 1872, well before the advent of formalin-fixation. At that time, the standard practice for fluid preservation of reptiles, amphibians, and fishes was direct preservation in “pure spirits of wine” (Günther, 1880). Thus, the holotype was most likely initially fixed in 90–100% ethanol (=Günther’s “pure spirits of wine”) and never exposed to formalin. Nevertheless, we opted to perform our initial DNA extraction using the methodology described in Hykin, Bi & McGuire (2015) for formalin-fixed tissues, with the goal being to perform an exome-capture experiment with this sample. The Hykin, Bi & McGuire (2015) procedure involves a series of initial ethanol washes followed by treatment in a heated alkali buffer solution to break cross-linkages before standard phenol-chloroform extraction. When this extraction returned a very low (potentially zero) yield, we performed a second pair of phenol-chloroform extractions involving phase-lock gel tubes followed by SPRI bead clean-up. This second round of extractions was performed with and without exposure to heated alkali solution. These extractions also failed to return sufficient DNA to move forward with library preparation. Despite minimal DNA yield, we made an attempt to PCR-amplify and sequence a short fragment of the mitochondrial ND2 gene from both DNA extracts. These experiments resulted in the amplification and sequencing of human ND2 in two separate experiments. Both of our low-yield DNA extractions were then sent to MYcroarray Inc. in Ann Arbor, MI where they were subjected to an extra silica purification designed for low-concentration fragment retention, and prepared as libraries. However, the library preparation retrieved only artifact and we did not proceed to targeted enrichment of selected exons or sequencing.

At this stage, we engaged with a lab specializing in genetic analysis of ancient DNA samples, with extraction and sequencing performed in a facility specifically designed for work with ancient samples (the Green/Shapiro Lab at UC Santa Cruz). No reptile work had previously been done in this facility and all work followed lab standards for working with historical samples (Fulton, 2011). The DNA extraction protocol was based on Dabney et al. (2013), Tin, Economo & Mikheyev (2014), and Cotoras et al. (2017)*.* An initial subsample of 40 mg of tissue was subdivided into ∼1 mm pieces and suspended in lysis buffer. The composition of the 100 mL lysis buffer aliquot was: 5.3 mL 1 M Tris-HCl (pH 8.0), 5.3 mL 0.2 M EDTA, 10.6 mL 20% Sarkosyl, 1 mL 2-mercaptoethanol, and 77.8 mL distilled water. The tissue was digested with a total of 1 mL of lysis buffer with 1 mg/mL proteinase K, initially incubated overnight at 56 °C, and then raised to 72 °C for 1 hr. The 72 °C incubation step was undertaken in case the sample had been exposed to formalin in order to reverse any potential crosslinks. Silica-based purification followed the centrifugation-based protocol described by Dabney et al. (2013). Briefly, 0.5 mL of 3 M sodium acetate was added to the lysate and them transferred to a tube with 13 mL of binding buffer. The binding buffer is prepared in a 50 mL tube by first adding 23.88 g of guanidine hydrochloride and then adding water to bring the volume to 30 mL. A key element of this purification protocol is the high salt concentration of this binding buffer, which enhances recovery of short DNA fragments. After complete dissolution of the guanidine hydrochloride, 25 µl of Tween-20 and sufficient isopropanol to bring the total volume to 50 mL were added. The mixture of sample, binding buffer, and sodium acetate was transferred into a Zymo extension reservoir attached to a MiniElute spin column. The spin column was then centrifuged for 10 min at 1,000 rpm, after which the spin column was transferred to a 1.5 mL Eppendorf tube. We performed a dry spin for 1 min at 13,000 rpm, followed by two washes with 750 µl of PE buffer (1 min spin at 6,000 rpm). To ensure the entire PE buffer was removed, we did a dry spin for 1 min at maximum speed. We eluted the purified extract in two volumes of 25 µl of TET. Each sample was centrifuged for 30 s at 13,200 RPM after 3–5 min of incubation. Because the elution displayed pigmentation, 25 µl of the extract was purified on a column filled with cross-linked polyvinylpyrrolidone (PVPP) (Arbeli & Fuentes, 2007). We also produced an extraction control consisting of lysis buffer that was subjected to the same set of procedures.

For genomic sequencing, we prepared three barcoded Illumina sequencing libraries (two for the holotype sample and one for the control) using the Meyer & Kircher (2010) protocol, starting with 5 µl of the PVPP purified DNA extraction. The same volume was used for the extraction control. The libraries were sequenced on an Illumina MiSeq machine using 150-cycle v3 chemistry (2 × 75). Following sequencing, adaptors were removed from reads and sequences were merged using SeqPrep2 (https://github.com/jeizenga/SeqPrep2). Default parameters were used with the exception of the following: -q 20 -L 30 -A AGATCGGAAGAGCACACGTC -B AGATCGGAAGAGCGTCGTGT. FastQC (http://www.bioinformatics.babraham.ac.uk/projects/fastqc/) confirmed that the sequence quality was good, with the normal base quality drop in the final five bases.

### (b) Sanger sequence data for *Draco fimbriatus* group specimens

Our team has generated a large number of complete ND2 sequences for *Draco* specimens, including for 65 exemplars representing the *D. fimbriatus* group. These sequences were available for comparison with ND2 sequence fragments obtained from the *D. cristatellus* holotype. PCR-amplification was undertaken using the primers Metf1 and ALAr2, with cycle sequencing involving these external primers plus the internal primers Metf5 and ND2r6 (see McGuire & Kiew, 2001 for details).

### (c) Exome-capture and screening of mitochondrial DNA

For another project, we generated an exome-capture data set using the MyBaits in-solution capture system for a set of 350 samples spanning all of *Draco*. This sample set included 14 *D. fimbriatus* group samples. The target loci for the exome capture include 1,400 exons and flanking sequences derived from transcriptome sequences (jointly representing 709 loci), which were supplemented with an additional 540 lizard-specific UCE loci. Libraries enriched for our target loci were barcoded and sequenced on an Illumina Hi Seq 4000. Although our experiment was specifically designed to avoid capturing mitochondrial genes, mitochondrial sequences are so abundant in genomic DNA extractions that some mitochondrial molecules inevitably find their way into the off-target by-catch (non-target DNA sequences that are obtained during an exome-capture experiment). We took advantage of this imperfect filter to obtain mitochondrial sequences for comparison with the *D. cristatellus* holotype. For TNHC 56763, we used Geneious version 8.1.7 (Kearse et al., 2012) to obtain a mostly complete representation of mitochondrial coding genes by mapping our raw exome capture data (including off-target sequences) to the complete mitochondrial genome of *Acanthasaura armata* available on GenBank (AB266452.1). A preliminary assessment of the identity of the sequences was performed with a BLAST search after collapsing duplicate sequences with fastx_collapser (http://hannonlab.cshl.edu/fastx_toolkit/commandline.html#fastx_ collapser_usage). The result of the BLAST search was visualized with the program MEGAN (Huson et al., 2007). Processed reads were mapped with BWA mem (Li & Durbin, 2009) against the reference partial mitochondrial genome of TNHC 56763. Duplicates were removed with samtools rmdup (Li et al., 2009). A total of 17 unique reads mapped against the reference after duplicate removal. They represent a total of 777 bp of the 8,114 bp reference. Most of the mapped regions had 1 × coverage and portions of four contigs had 2 × coverage. The average length of the mapped reads was 53 bp. Finally, for each of the 14 *D. fimbriatus* group samples included in our exome-capture experiment, we used Geneious to map our raw sequencing reads to 10 mitochondrial contigs obtained for the *D. cristatellus* holotype. The raw sequence data is available on the SRA database.

### (d) Analysis of DNA sequence variation

Our analysis of DNA sequence variation included alignment of homologous DNA sequences and a simple count of nucleotide base substitutions between the *Draco cristatellus* holotype, sample TNHC 56763 from Santubong, Sarawak, and our selection of *D. fimbriatus* and *D. punctatus* samples from the Malay Peninsula, Sumatra, the Mentawai Islands, Java, and Borneo. Specimens examined are listed in Table 1. We also performed a heuristic parsimony analysis to obtain a phylogram for the *D. fimbriatus* group and performed a non-parametric bootstrap analysis with 1,000 replicates to assess branch support. We did not perform a more rigorous maximum likelihood or Bayesian analysis because our primary objective was to assess uncorrected relative branch lengths. Phylogenetic analyses were performed in PAUP version 4 (Swofford, 2002).

**Table 1:** Numbers of base substitutions and sequence divergence values between the *Draco cristatellus* holotype and 14 exemplars representing the *D. fimbriatus* group. Base pair differences between the *Draco cristatellus* holotype and each of 14 *D. fimbriatus* group samples for six mitochondrial genes. For ND2, the data used for comparisons were generated using standard Sanger sequencing. For all other genes, the data were derived from exome-capture off-target sequences. Mean sequence divergence values relative to the holotype are provided for each gene for each species. The final column indicates the total number of base changes and percentage divergences from the holotype, with the caveat that these summary values do not account for the unique evolutionary rates that typify each of these mitochondrial genes.

	COX III	COI	COI	ND4L	ND5	ND2	Total (%)
	contig3a	contig12a	contig12b	contig13	contig15	Sanger	
*cristatellus* TNHC 56763	0/43 (0%)	1/79 (1.3%)	1/81 (1.2%)	0/79 (0%)	0/64 (0%)	3/183 (1.6%)	5/547 (0.9%)
*punctatus* Borneo TNHC 56766		7/79 (8.9%)	7/69 (10.1%)			30/183 (16.4%)	44/331 (13.3%)
*punctatus* Borneo TNHC 56764		6/79 (7.6%)	8/81 (9.9%)	10/69 (14.5%)		28/183 (15.3%)	52/412 (12.6%)
*punctatus* Malay Pen. LSUHC 5617	5/43 (11.6%)			10/79 (12.7%)		32/183 (17.5%)	47/305 (15.4%)
*punctatus* Mentawai MVZ 270632	4/43 (9.3%)	10/79 (12.7%)	7/81 (8.6%)	12/79 (15.2%)		37/183 (20.2%)	70/465 (15.1%)
*punctatus* Batu Ids MVZ 270636	4/43 (9.3%)	10/79 (12.7%)	7/81 (8.6%)	12/79 (15.2%)		35/183 (19.1%)	68/465 (14.6%)
*punctatus* Sumatra MVZ 270835	4/43 (9.3%)	9/79 (11.4%)	7/81 (8.6%)	11/79 (13.9%)		35/183 (19.1%)	66/465 (14.2%)
*punctatus* Banyak Ids MVZ 270829	4/43 (9.3%)	10/79 (12.7%)	7/81 (8.6%)	9/59 (15.3%)		37/183 (20.2%)	67/465 (14.4%)
*fimbriatus* Mal Pen TNHC 57954	4/43 (9.3%)	7/71 (9.9%)	7/81 (8.6%)	9/79 (11.4%)	12/64 (18.8%)	29/183 (15.8%)	68/521 (13.1%)
*fimbriatus* Mal Pen TNHC 58565	4/43 (9.3%)	10/79 (12.7%)	7/80 (8.8%)	9/79 (11.4%)	12/64 (18.8%)		42/345 (12.2%)
*fimbriatus* Sumatra MZB Lace.14276		9/79 (11.4%)	5/56 (8.9%)	8/79 (10.1%)		32/183 (17.5%)	54/397 (13.6%)
*fimbriatus* Sumatra MVZ 239473		9/79 (11.4%)	8/81 (9.9%)	8/79 (10.1%)	11/64 (17.2%)		36/303 (11.9%)
*hennigi* LSUMZ 81446		11/79 (13.9%)		8/79 (10.1%)	12/64 (18.8%)	23/183 (12.6%)	54/405 (13.3%)
*hennigi* LSUMZ 81447		11/79 (13.9%)		8/79 (10.1%)	13/64 (20.3%)	23/183 (12.6%)	55/405 (13.6%)
						*cristatellus*	5/547 (0.9%)
						*punctatus*	414/2908 (14.2%)
						*fimbriatus*	200/1566 (12.8%)
						*hennigi*	90/700 (12.9%)

### (e) Data availability and Permits

Sanger sequence data are available at GenBank (SRP135680) and a matrix for the mitochondrial ND2 gene for the *D. fimbriatus* group is included as supplemental materials. This research was undertaken in accordance with UC Berkeley Animal Use Protocol Number AUP-2014-12-6954. Fieldwork was undertaken with research permits issued by the Economic Planning Unit of Malaysia (UPE:40/200/19 SJ.363) and the Indonesian Institute of Sciences (LIPI: No. 2411/FRP/SM/X/2008 and No. 0115/FRP/SM/VI/2009).

## Results

The MiSeq run generated a total of 1,086,926 paired-end reads after combining the data from two different libraries (prepared identically) from the same extract. Most of the fragments on the original library were 75 bp, whereas the average length of the mapped reads was 53 bp. Raw sequences were merged if possible and duplicates collapsed, producing a total of 538,995 reads, which were BLAST searched (Altschul et al., 1990) against the NCBI database. 115,266 reads were successfully assigned, and of these, 47,400 hits corresponded to bacteria, 40,751 were assigned to mammals (of which 29,720 were specifically assigned to human), 334 were assigned to Reptilia, and 48 were specifically assigned to *Anolis carolinensis*. Only three reads were assigned to *Draco*, each of which involved the mitochondrial ND2 sequence posted on GenBank for *Draco cristatellus* sample TNHC 56763 (see below). The paucity of *Draco* hits is not surprising given that there are no *Draco* reference genomes to map to. The extraction control was sequenced producing a total of 104,417 PE reads. After processing (adaptor removal, merged if possible, and duplicate removal) a total of 15,480 reads were assigned by the BLAST search. Bacteria were represented by 6,805 reads, mammals by 4,711 reads (of those, 3103 were assigned to human), and reptiles were assigned no reads (one read was assigned to chicken, *Gallus gallus*).

Of the three holotype ND2 reads, two were broadly overlapping, and the joint ND2 data obtained from the holotype totaled only 125 bp. To search for additional mitochondrial contigs in the holotype MiSeq data, we first generated a partial mitochondrial genome for TNHC 56763 from exome-capture off-target by-catch, which returned 8,114 bp of protein-coding gene sequence data. Mapping the holotype data to the TNHC 56763 mitochondrial assembly resulted in the recovery of an additional 17 reads totaling 777 bp of mitochondrial sequence data representing six genes (COI, COXIII, ATPase8, ND4, ND4L, ND5). Thus, a total of 13 contigs were assembled from 20 reads with an average length of 61 bp. Most of the mapped regions had 1 × coverage and parts of four contigs had 2 × coverage. No reads were recovered when mapping the extraction blank against the same reference. In comparing the holotype sequence data with TNHC 56763 across the 721 bp of homologous sequence data, we found that the two samples were only weakly divergent from one another, sharing the same base calls at 710 of 721 positions for a raw sequence divergence of 1.5%. We then mapped the raw reads from the exome captures for the remaining 13 *D. fimbriatus* group samples to the 10 *D. cristatellus* holotype contigs, which returned as few as two and as many as six homologous sequences per sample.

Comparison of the mitochondrial data obtained from the *Draco cristatellus* holotype with homologous data obtained for *D. fimbriatus* group samples found that the sample TNHC 56763 from Santubong, Sarawak, Malaysian Borneo was much more similar to the holotype than were any other *D. fimbriatus* group samples. When limiting our comparison to the six gene fragments for which we had between six and 13 corresponding sequences for comparison to the holotype, we found that TNHC 56763 was 0.9% divergent from the holotype, whereas all other samples ranged between 11.9% and 15.1% divergent. TNHC 56763 differed from the holotype at just five of 547 base positions, whereas the other samples differed from the holotype at from 36 of 303 bp to 70 of 465 base positions. The ND2 comparisons were most comprehensive because we had access to complete ND2 sequences for 65 *D. fimbriatus* group samples. Whereas TNHC 56763 differed from the holotype at three of 183 base positions, all other samples differed by at least 24 base positions. Notably, the two *D. punctatus* samples from Sarawak (the type locality for *D. cristatellus*) differed from the holotype at 28 and 30 of 183 base positions (15.3% and 16.4%, respectively).

**Figure 1:**
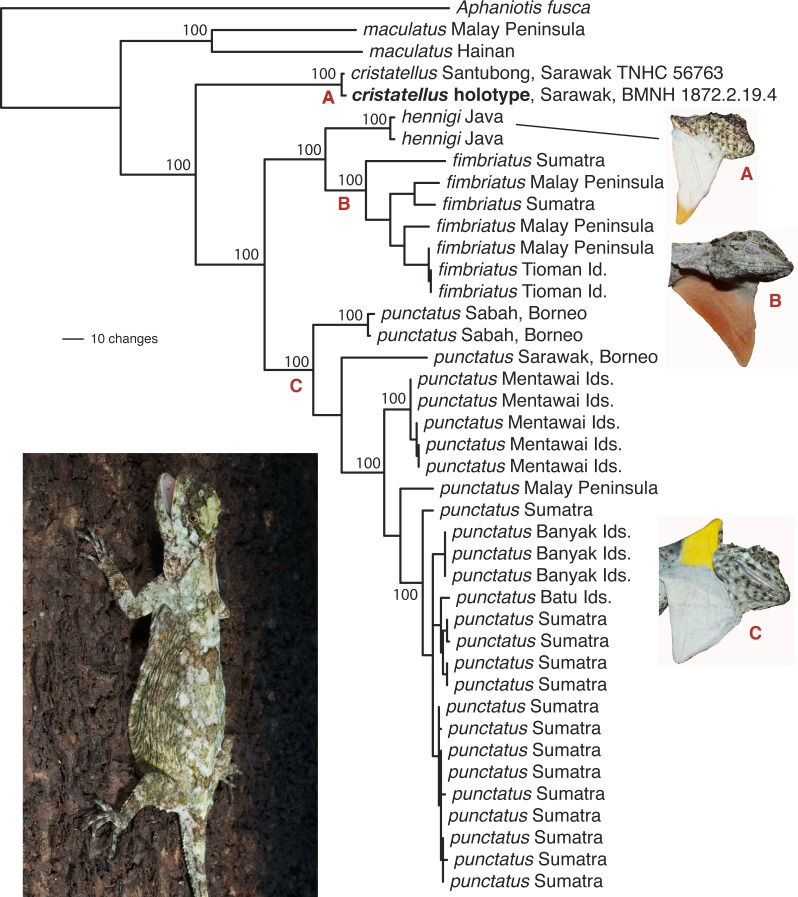
Phylogenetic tree for the *Draco fimbriatus* group including the *D. cristatellus* holotype. Phylogenetic tree for the *Draco fimbriatus* group based on a parsimony analysis of the complete mitochondrial ND2 gene (1,032 bp). The *D. cristatellus* holotype includes 183 bp of sequence data. Only two of 28 available *D. maculatus* samples were included to simplify the image. Non-parametric bootstrap values (1,000 replicates) are superimposed on the single most parsimonious phylogram for select clades. The photo in the bottom left is *Draco punctatus*. Photo: Jimmy A. McGuire.

A parsimony phylogenetic analysis of the ND2 gene including the *D. cristatellus* holotype strongly supports the monophyly of the holotype together with TNHC 56763 to the exclusion of all other *D. fimbriatus* group samples with 100% bootstrap support (Fig. 1). Further, the 1.6% ND2 sequence divergence between TNHC 56763 and the holotype is only slightly greater than that between samples of *D. punctatus* from a single locality (Sitahuis, Sumatra; uncorrected ND2 divergence of 1.2%). This does not consider the possibility that one or more of the five documented base substitutions could be sequencing errors resulting either from damage to the holotype DNA or random errors in our low coverage data, as we could not apply informatics pipelines developed to identify post-mortem damage of ancient DNA to our data (e.g., Mateiu & Rannala, 2008; Molak & Ho, 2011). Notably, one of the three inferred ND2 substitutions is a first position C->T change that would result in a proline to serine amino acid replacement, suggesting that this might be the result of post-mortem deamination of the template molecule or sequencing error.

## Discussion

In the present study, we applied HTS to a 145-year old fluid-preserved holotype specimen in an effort to disentangle an otherwise intractable taxonomic question. The problem stems from the fact that one of the species in the *Draco fimbriatus* group, *Draco cristatellus*, was described using limited color information, and because fluid-preserved specimens representing multiple sympatric *D. fimbriatus* group species are often indistinguishable from one another without color information. Indeed, sympatric species in this complex are effectively cryptic once they have been prepared as museum specimens. This combination of circumstances rendered it virtually impossible to resolve the species status of *D. cristatellus* relative to *D. punctatus* and *D. fimbriatus,* two widespread species on the Greater Sunda Shelf. Importantly, a sample (TNHC 56763) collected in 1996 by JAM provides phylogenetic evidence for a third *D. fimbriatus* group species on Borneo, with the natural question being whether this sample is conspecific with the name-bearing holotype specimen of *D. cristatellus* housed in the British Museum of Natural History. Several species composition outcomes were possible, all of which were considered in a hypothesis-testing framework. In Hypothesis 1, *D. cristatellus* and *D. punctatus* are synonyms, together representing a single species distinct from *D. fimbriatus* and TNHC 56763. In Hypothesis 2, *D. cristatellus* and *D. fimbriatus* are synonyms. In Hypothesis 3, *D. cristatellus* is a species distinct from *D. punctatus* and *D. fimbriatus* but conspecific with TNHC 56763. Finally, in Hypothesis 4, *D. cristatellus*, *D. fimbriatus*, *D. punctatus*, and TNHC 56763 all represent distinct species, with TNHC 56763 representing a fourth sympatric species on Borneo. The only way to conclusively test these alternative hypotheses was to obtain informative genetic data from the holotype specimen of *D. cristatellus* for comparison with TNHC 56763, and representative specimens of *D. fimbriatus* and *D. punctatus*.

We initially believed that genetic data would easily be retrieved from the *Draco cristatellus* holotype. The holotype was prepared before the advent of formalin-fixation, and we consequently had reason to believe that the specimen was originally fixed with ethanol and had never been exposed to formalin. Because tissue samples collected for genetic analysis are routinely stored in ethanol, we were confident that the holotype would still hold high molecular-weight DNA suitable for genomic sequencing. Our hope was to perform exome-capture with this sample and include it in a larger *Draco* phylogenomic data set. However, our initial attempts at extracting DNA from the sample using methods appropriate for historical and formalin-fixed tissues failed, forcing us to adjust both our approach and our expectations. Fortunately, our alternative hypotheses proved testable without comprehensive genomic data from the holotype. Indeed, analysis of the initial 125 bp of mitochondrial ND2 data identified when the holotype sequence data was subjected to GenBank BLAST allowed us to reject hypotheses 1, 2, and 4 in favor of Hypothesis 3. The additional 596 bp of mitochondrial data obtained via mapping of holotype contigs to the reconstructed mitochondrial genome for TNHC 56763 simply provided additional confirmation that *D. cristatellus* and *D. punctatus* are each valid species, and that our specimen TNHC 56763 from Santubong, Sarawak is indeed a true *D. cristatellus* exemplar. This finding was a best-case scenario because all future specimens for which tissue samples are obtained can now be compared with known *D. cristatellus*, *D. punctatus*, and *D. fimbriatus* samples for genetic identification.

What lessons can be learned from our attempt to obtain genetic data from the *Draco cristatellus* holotype? First, even when initial attempts at extraction and quantification of DNA suggest that none is present, small numbers of DNA molecules may survive in the sample. For questions of simple species identification involving old and highly degraded samples, it may only be necessary to obtain limited data—even a few hundred base pairs of mitochondrial data may be sufficient to address the question. Our study shows that this is indeed possible even when initial assessments suggest that DNA in a tissue sample has been highly degraded. Obtaining data in these instances will likely require highly specialized extraction procedures such as the silica-column based extraction methodology utilized here, followed by short-fragment sequencing. Finally, we believe that the difficulty we confronted with this ethanol-fixed sample—which is consistent with the problems experienced by Ruane & Austin (2016) with their presumed alcohol-fixed samples (four of five of which failed to sequence)—suggests that older ethanol-fixed specimens might be more problematic than formalin-fixed specimens for genomic sequencing efforts (see Handt et al., 1994 for a description of DNA hydrolysis). We note that tissue samples are routinely stored in 95% ethanol for future DNA sequencing, and that standard dogma is that old museum samples fixed in ethanol should be readily amenable to DNA sequencing, whereas formalin-fixed samples are expected to be particularly challenging for sequencing efforts (see Zimmermann et al., 2008). Thus, it would be both surprising and ironic if old museum specimens originally fixed and stored in ethanol prove to be less favorable for HTS than formalin-fixed specimens. This possibility has important implications for curatorial practices. Not only is potential hydrolytic damage cause for concern with whole fluid specimens stored in ethanol, but it could also be problematic for tissue samples stored in ethanol that are not maintained in sub-zero degree conditions. Evaporation of ethanol from tissue vials might render even modern tissue samples problematical for genetic analysis.

## Conclusions

The development of HTS has revolutionized biological research by making genome-scale data readily available at a reasonable cost, even for non-model organisms. Systematists have fully embraced these advances in data acquisition for freshly sampled specimens, but are just beginning to harness HTS for the millions of fluid-preserved historical samples housed in natural history collections around the world. As we have shown here, acquiring genetic data from old museum specimens will sometimes present special challenges, but the information that can be gleaned from such specimens may be the only way to conclusively resolve previously intractable evolutionary and taxonomic questions.

##  Supplemental Information

10.7717/peerj.4470/supp-1 Supplemental Information 1 ND2 sequence data for members of the *Draco fimbriatus* group plus 13 mitochondrial sequence reads obtained from the *D. cristatellus* holotype This file includes ND2 sequence data obtained via traditional Sanger sequencing for 39 individuals representing *Draco cristatellus*, *D. fimbriatus*, *D. hennigi*, *D. punctatus*, and *D. maculatus*, plus 183 base pairs of ND2 obtained via MiSeq HTS for the *Draco cristatellus* holotype (BMNH 1872.2.19.4). In addition, the 13 mitochondrial sequence reads obtained for the *D. cristatellus* holotype are provided.

## Additional Information and Declarations

### Field Study Permissions

The following information was supplied relating to field study approvals (i.e., approving body and any reference numbers): Specimens collected in support of this research were obtained under research permits issued by the Economic Planning Unit (EPU) of Malaysia and the Indonesian Institute of Sciences (LIPI).

### DNA Deposition

The following information was supplied regarding the deposition of DNA sequences: The sequence data is available at GenBank (SRP135680) and in the Supplemental Information.

### Data Availability

The following information was supplied regarding data availability: The sequence data is available at GenBank (MH070349–MH070383; SRP135680) and in the Supplemental Information.

## References

[ref-1] Altschul SF, Gish W, Miller EW, Myers W, Lipman DJ (1990). Basic local alignment search tool. Journal of Molecular Biology.

[ref-2] Arbeli Z, Fuentes CL (2007). Improved purification and PCR amplification of DNA from environmental samples. FEMS Microbiology Letter.

[ref-3] Boulenger GA (1900). Descriptions of new batrachians and reptiles from the Larut Hills, Perak. Annals and Magazine of Natural History, Series 7.

[ref-4] Cotoras DD, Murray J, Kapp RG, Gillespie C, Griswold WB, Simison RE, Green GGR, Shapiro B (2017). Ancient DNA resolves the history of *Tetragnatha* (Araneae, Tetragnathidae) spiders on Rapa Nui. Genes.

[ref-5] Dabney J, Knapp M, Glocke I, Gansauge M-T, Weihmann A, Nickel B, Valdiosera C, Garcia N, Paabo S, Arsuaga J-L, Meyer M (2013). Complete mitochondrial genome sequence of a Middle Pleistocene cave bear reconstructed from ultrashort DNA fragments. Proceedings of the National Academy of Sciences of the United States of America.

[ref-6] De Rooij N (1915). The reptiles of the Indo-Australian archipelago. I Lacertilia, Chelonia, Emydosauria.

[ref-7] Fulton TL, Shapiro M, Hofreiter B (2011). Setting up an ancient DNA laboratory. Ancient DNA methods and protocols.

[ref-8] Grandison AGC (1972). The Gunong Benom expedition 1967. 5. Reptiles and amphibians of Gunong Benom with a description of a new species of *Macrocalamus*. Bulletin of the British Museum (Natural History).

[ref-9] Günther A (1872). On the reptiles and amphibians of Borneo. Proceedings of the Zoological Society of London.

[ref-10] Günther A (1880). An introduction to the study of fishes.

[ref-11] Handt O, Höss M, Krings M, Pääbo S (1994). Ancient DNA: methodological challenges. Experientia.

[ref-12] Hennig W (1936). Revision der Gattung *Draco* (Agamidae). Temminckia.

[ref-13] Huson DH, Auch AF, Qi J, Schuster SC (2007). MEGAN analysis of metagenomic data. Genome Research.

[ref-14] Hykin SM, Bi K, McGuire JA (2015). Fixing formalin: a method to recover genomic-scale DNA sequence data from formalin-fixed museum specimens using high-throughput sequencing. PLOS ONE.

[ref-15] Inger RF (1983). Morphological and ecological variation in the flying lizards (genus *Draco*). Fieldiana Zoology, New Series.

[ref-16] Kearse M, Moir R, Wilson A, Stones-Havas S, Cheung M, Sturrock S, Buxton S, Cooper A, Markowitz S, Duran C, Thierer T, Ashton B, Meintjes PL, Drummond AJ (2012). Geneious basic: an integrated and extendable desktop software platform for the organization and analysis of sequence data. Bioinformatics.

[ref-17] Li H, Durbin R (2009). Fast and accurate short read alignment with Burrows-Wheeler Transform. Bioinformatics.

[ref-18] Li H, Handsaker B, Wysoker A, Fennell T, Ruan J, Homer N, Marth G, Abecasis G, Durbin R, 1000 Genome Project Data Processing Subgroup (2009). The Sequence alignment/map (SAM) format and SAMtools. Bioinformatics.

[ref-19] Manthey U (2008). Agamid lizards of Southern Asia. Draconinae 1.

[ref-20] Mateiu L, Rannala B (2008). Bayesian inference of errors in ancient DNA caused by postmortem degradation. Molecular Biology and Evolution.

[ref-21] McGuire JA (2003). Allometric prediction of locomotor performance: an example from Southeast Asian flying lizards. American Naturalist.

[ref-22] McGuire JA, Alcala AC (2000). A taxonomic revision of the flying lizards of the Philippine Islands (Iguania: Agamidae: *Draco*), with a description of a new species. Herpetological Monographs.

[ref-23] McGuire JA, Brown RM, Mumpuni, Riyanto A, Andayani N (2007). The flying lizards of the *Draco lineatus* group (Squamata: Iguania: Agamidae): a taxonomic revision with descriptions of two new species. Herpetological Monographs.

[ref-24] McGuire JA, Kiew BH (2001). Phylogenetic systematics of Southeast Asian flying lizards (Iguania: Agamidae: *Draco*) as inferred from mitochondrial DNA sequence data. Biological Journal of the Linnean Society.

[ref-25] Meyer M, Kircher M (2010). Illumina sequencing library preparation for highly multiplexed target capture and sequencing. Cold Spring Harbor Protocols.

[ref-26] Molak M, Ho SYW (2011). Evaluating the impact of post-mortem damage in ancient DNA: a theoretical approach. Journal of Molecular Evolution.

[ref-27] Musters CJM (1983). Taxonomy of the genus * Draco* L. (Agamidae, Lacertilia, Reptilia). Zoologische Verhandelingen.

[ref-28] Palkopoulou E, Mallick S, Skoglund P, Enk J, Rohland N, Li H, Omrak A, Vartanyan S, Poinar H, Götherström A, Reich D, Dalén L (2015). Complete genomes reveal signatures of demographic and genetic declines in the woolly mammoth. Current Biology.

[ref-29] Prüfer K, Racimo F, Patterson N, Jay F, Sankararaman S, Sawyer S, Heinze A, Renaud G, Sudmant PH, De Filippo C, Li H, Mallick S, Dannemann M, Fu1 Q, Kircher M, Kuhlwilm M, Lachmann M, Meyer M, Ongyerth M, Siebauer M, Theunert C, Tandon A, Moorjani P, Pickrell J, Mullikin JC, Vohr SH, Green RE, Hellmann I, Johnson PLF, Blanche H, Cann H, Kitzman JO, Shendure J, Eichler EE, Lein ES, Bakken TE, Golovanova LV, Doronichev VB, Shunkov MV, Derevianko AP, Viola B, Slatkin M, Reich D, Kelso J, Pääbo S (2014). The complete genome sequence of a Neandertal from the Altai Mountains. Nature.

[ref-30] Ruane S, Austin CC (2016). Phylogenomics using formalin-fixed and 100+ year-old intractable natural history specimens. Molecular Ecology Resources.

[ref-31] Swofford DL (2002).

[ref-32] Tin MM-Y, Economo EP, Mikheyev AS (2014). Sequencing degraded DNA from non-destructively sampled museum specimens for RAD-tagging and low-coverage shotgun phylogenetics. PLOS ONE.

[ref-33] Uetz P (2006). The EMBL reptile database. http://www.reptile-database.org.

[ref-34] Zimmermann J, Hajibabaei M, Blackburn DC, Hanken J, Cantin E, Posfai J, Evans Jr TC (2008). DNA damage in preserved specimens and tissue samples: a molecular assessment. Frontiers in Zoology.

